# The Implementation of the Advocacy Intervention for Midlife and Older Women Who Have Experienced Intimate Partner Violence: Protocol for a Randomized Controlled Trial

**DOI:** 10.2196/57886

**Published:** 2024-10-30

**Authors:** Lori Weeks, Kathleen Allen, Catherine Holtmann, Joni Leger, Suzanne Dupuis-Blanchard, Colleen MacQuarrie, Marilyn Macdonald, Elaine Moody, Christie Stilwell, Heather Helpard, Danie Gagnon

**Affiliations:** 1 School of Health Administration Dalhousie University Halifax, NS Canada; 2 Muriel McQueen Fergusson Centre for Family Violence Research University of New Brunswick Fredericton, NB Canada; 3 Muriel McQueen Fergusson Centre for Family Violence Research, Department of Sociology University of New Brunswick Fredericton, NB Canada; 4 School of Nursing, Centre for Aging Research Université de Moncton Moncton, NB Canada; 5 Department of Psychology University of Prince Edward Island Charlottetown, PE Canada; 6 School of Nursing Dalhousie University Halifax, NS Canada; 7 Faculty of Health Dalhousie University Halifax, NS Canada; 8 Rankin School of Nursing St. Francis Xavier University Antigonish, NS Canada

**Keywords:** intimate partner violence, intervention, virtual, midlife, aging

## Abstract

**Background:**

Midlife and older women who experience intimate partner violence (IPV) often have less access to supports and services than younger women. There is far less focus on research and supports for midlife and older women compared to younger women experiencing IPV, and often, neither elder abuse nor IPV services meet their needs. Few interventions are available to meet the needs of midlife and older women.

**Objective:**

The goal of this randomized controlled trial is to test the effectiveness of an advocacy intervention for midlife and older women who experience IPV and to learn from the experiences of those who implement and participate in the program.

**Methods:**

This trial is a 2-arm, unblinded, parallel, pragmatic randomized controlled trial with a qualitative component. Eligible participants will be women who live in the Maritime provinces of Canada (New Brunswick, Nova Scotia, and Prince Edward Island), who are in midlife and older (aged approximately ≥50 years), and who are currently in a relationship with an abusive partner or have recently left an abusive partner. Facilitators will be trained to deliver the intervention. The intervention will be entirely virtual and will consist of 2 components: (1) an empowerment component, which will involve sharing resources and information with the women; and (2) a social support component, which will include providing support and encouragement to women for 12 weeks. Quantitative effectiveness data will be collected from all trial participants at baseline, 3 months after the intervention, and 9 months after the intervention about the incidence and severity of IPV, physical and mental health, and safety behaviors and strategies. Qualitative interviews will be conducted with the facilitators and intervention group participants. Control group participants will receive a static, nontailored version of the advocacy intervention for midlife and older women (AIM) intervention materials after baseline data collection.

**Results:**

A total of 12 facilitators have been trained to deliver the AIM intervention to trial participants. Participant recruitment and data collection will be completed in January 2025. Data analysis will continue throughout the data collection period, and the results will be disseminated by December 2025.

**Conclusions:**

This research will result in the adaptation and testing of a program to support and empower midlife and older women in the Maritime provinces of Canada who experience IPV.

**Trial Registration:**

International Standard Randomized Controlled Trial Registry ISRCTN30646991; https://doi.org/10.1186/ISRCTN30646991

**International Registered Report Identifier (IRRID):**

DERR1-10.2196/57886

## Introduction

### Background and Rationale

Intimate partner violence (IPV) is widely recognized as a major public health concern globally [1] and in Canada, with 1 in every 10 adults aged ≥65 years experiencing some form of abuse each year [2]. We use the term IPV to refer to any type of abusive behavior (eg, emotional, verbal, physical, sexual, or financial) that occurs between intimate partners, such as spouses, those living in a common-law relationship, or in a dating relationship. While our knowledge is far less complete on IPV among older versus younger women, a mistaken assumption is that IPV ceases with age [3]. Prevalence studies have shown that between 15% and 30% of older women report IPV at some point in their lives [4-7]. Incidence studies have shown that 8.6% of women currently in partnerships experienced IPV since reaching the age of 55 years [8]; 3.5% of women aged ≥65 years experienced IPV in the past 5 years, with 2.2% in the past year [4]; and 5.5% of women aged between 50 and 64 years experienced IPV in the past 2 years [9]. In a study of coroners’ files of homicide by individuals aged ≥65 years, 89% of homicide survivors were female, and of these, 93% were current or former spouses of male perpetrators [10]. It is generally recognized that IPV among older women is underestimated.

There is far less focus on research and supports for midlife and older women compared to younger women experiencing IPV, and often, neither elder abuse nor IPV services address their unique needs. Researchers define midlife as a pivotal time in the life span, representing the transition from earlier to later periods of life during which individuals are balancing multiple roles, life transitions, and opportunities and challenges [11]. Midlife is generally viewed as spanning from the ages of 40 to 60 years [11]. Midlife is viewed as a bridge between generations and can involve multiple caregiving responsibilities (eg, grandchildren, adult children, and aging parents), career development and retirement planning, changes in family leadership, and changes in health status (eg, developing chronic illnesses) [11]. During midlife, women often juggle multiple demands. People providing elder abuse services often are not equipped to support women from a gendered and relational lens [12], and those providing family violence services often focus on meeting the needs of girls, young women, and women with dependent children [13]. Thus, the needs of midlife and older women who experience IPV may not be met by the assistance of those working in elder abuse or IPV services. In addition, initiatives that offer IPV support and services to older women in Canada do not necessarily implement formal program evaluations, and therefore, information about the effectiveness of these initiatives is lacking [14].

Women have unique needs and experiences and require individualized help to receive the support they need. In a recent research study conducted by our team, we interviewed midlife and older women and found many groups of women, including francophone women, new immigrants (eg, from Arabic-speaking countries), Black women, and people living in rural places with limited access to services, have unique needs [15]. Research suggests that other populations considered vulnerable in Canada are also at increased risk of experiencing IPV, such as Indigenous women, gender and sexual minority groups, and people living with disabilities [16]. Women considered vulnerable and women with multiple vulnerable identities are at an increased risk of experiencing IPV due to them being exposed to multiple forms of systemic oppression (eg, racism, agism, and ableism), making it difficult to break the cycle of violence [17].

Given that many existing services are not appropriate to meet the unique needs of older women, there is an emergent emphasis on supports that do address the unique preferences and situations, or “victim-centered supports” [18,19]. There are few published studies about initiatives focused specifically on IPV and older women [14]. While a feminist lens is often used in research and with practitioners working with abused women and girls, gender-based analysis is often neglected by scholars and practitioners working with or for abused midlife and older women. In this research, we will use an intersectional approach to consider how various social factors (eg, age, gender, language, race, ethnicity, culture, and geographic region) intersect to impact women’s risk of IPV exposure and their experiences of violence, their ability to seek help and access services, as well as their overall health and quality of life [20].

### Objectives

The goal of this randomized controlled trial (RCT) with a qualitative component is to test the effectiveness of the 2 components of an advocacy intervention for midlife and older women (AIM) that has been adapted to support midlife and older women who have experienced IPV and to learn from the experiences of those who will implement and participate in the program. This research will support the adaptation, scale-up, and spread of the program to support and empower women in Canada in midlife and older who experience IPV to improve their health and quality of life.

## Methods

### Trial Design

**Figure 1:**
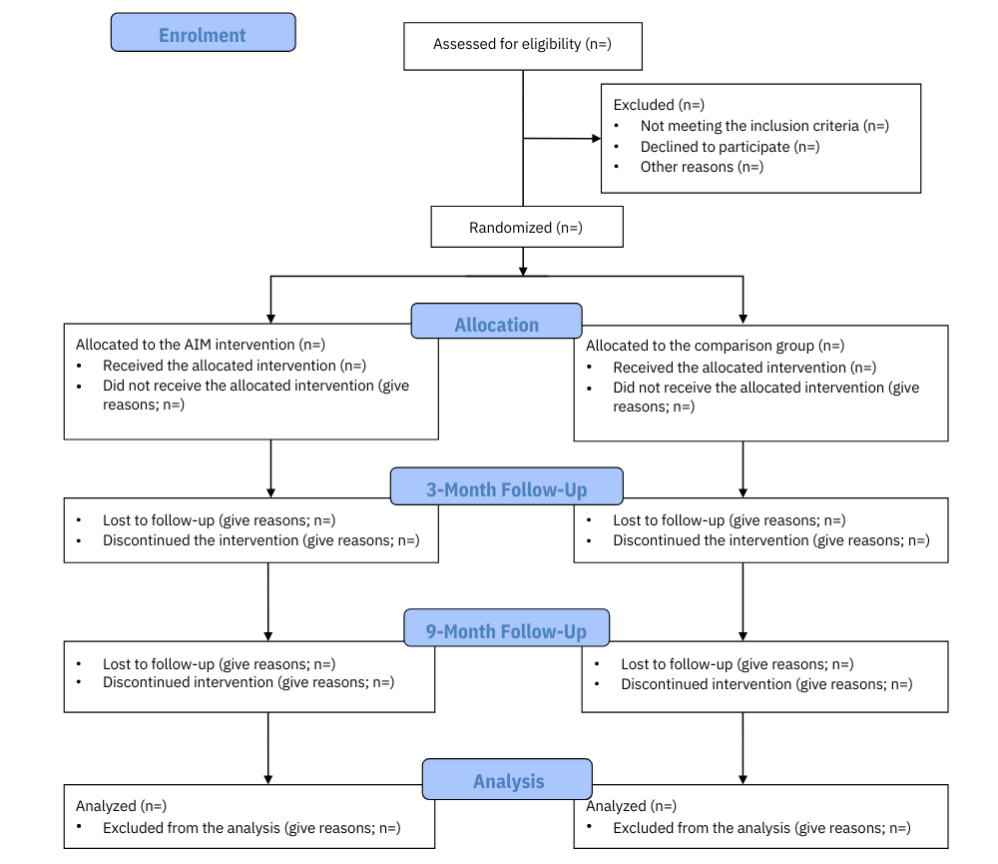
CONSORT (Consolidated Standards of Reporting Trials) flow diagram.

This trial is a 2-arm, unblinded, parallel, pragmatic RCT [21] registered in the International Standard Randomized Controlled Trial Registry (ISRCTN30646991). This protocol is reported according to the SPIRIT (Standard Protocol Items: Recommendations for Interventional Trials) guidelines [22]. The intervention has been modeled using the Template for Intervention Description and Replication checklist [23], and the results will be reported according to the CONSORT (Consolidated Standards of Reporting Trials) statement for RCTs [24]. Figure 1 displays the CONSORT flow diagram for this RCT.

### Study Setting

Participants will be recruited from the 3 Maritime provinces of Canada (New Brunswick, Nova Scotia, and Prince Edward Island). As the program will be delivered virtually, we are not restricted to a certain number of sites. Our rationale for including the Maritime provinces for this study is that the members of our research team are located within the Maritime provinces, and we have extensive professional and personal networks that will help to support the success of the research. The Maritime provinces are among the Canadian provinces with the highest proportion of adults aged ≥65 years [25]. In addition, New Brunswick is the only Canadian province that is officially bilingual and represents a unique opportunity to include both anglophone and francophone participants from the same geographic region [26].

### Eligibility Criteria

The following inclusion criteria will be used to determine if women are eligible to be enrolled in the trial. The potential participants are those who self-identify as a woman; are in midlife and older (aged approximately ≥50 years); are currently living in the Maritime provinces; can participate in an interview in either English or French; and are currently in a relationship with an abusive partner or have recently left an abusive partner within the last year, including a same-sex partner.

In a recent systematic review [27], our team used the theoretical concepts of reclaiming self-theory and the four levels of prevention [28-31] to classify IPV interventions into the categories of (1) preventing IPV, (2) identifying and documenting IPV, (3) supporting women while living with or leaving an abusive partner, and (4) supporting women after leaving an abusive partner. The intervention used in this trial will target women in category 3 as it is designed to empower women to make decisions and take action related to the abuse they are experiencing or have recently experienced.

The dynamics of abusive relationships are such that leaving an abusive partner is often a process that involves leaving and returning multiple times [32]. During this process, the woman may reach out to available services, such as outreach services, or live in a transition house or other form of safe housing. Thus, the participants may have already contacted IPV services and may be using various forms of services or initiatives during the course of this study. Given that our research team will not be interfering with the supports and services that the participants are already using during their participation in the study, this study aligns with the tenets of a pragmatic trial [33]. Our team has had success recruiting older women experiencing IPV who had already accessed services, but we have had challenges recruiting older women in the region who have never contacted an organization for support [14]. The intervention is designed to provide information about other resources and supports available to women and to encourage women to use these services; therefore, it is not a concern that the women access and use other supports during our study.

### AIM Intervention

#### Overview

After completing a recent systematic review focused on the IPV interventions relevant to women during the COVID-19 pandemic, including research evidence from 4 systematic reviews and 20 individual studies [27], our team identified an advocacy health promotion program [34] that was ideal to adapt, implement, and test in this RCT. Given that the advocacy health promotion program is not a proprietary program, permission was not required to adapt it for our research. The intervention reported by Tiwari et al [34] has been selected to be adapted for this trial because the researchers reported that it significantly reduced depression and psychological aggression scores and improved perceived social support and the use of safety-promoting behaviors. Other interventions that we examined in our systematic review either did not result in positive outcomes for the health and safety needs of women or the results did not reach statistical significance. We also found that providing highly tailored interventions can contribute to positive outcomes [35,36], and the intervention reported by Tiwari et al [34] could be tailored as needed. This study will pay particular attention to ensuring that those who are trained to deliver the intervention provide individualized and person-centered support.

An innovative aspect of this research is the adaptation of the intervention to meet the needs of midlife and older women that can often be different from those of younger women. For example, leaving one’s home, property, and community may not be a priority for older women experiencing IPV [19], and therefore, the women will be provided support to empower them to identify their own goals and strategies for achieving those goals. The intervention has also been adapted to meet the needs of women who reside in the Maritime provinces of Canada. Information on local IPV services and resources will be provided to the participants. The format will also be adapted to meet the preferences of the women to use whatever form of technology they prefer for the social support component of the study. In addition, those trained to deliver the program will use a person-centered perspective to support the unique needs of the participants, such as for participants who are francophone, living in a rural place, or a new immigrant to Canada.

We have chosen to name the program the Advocacy Intervention for Women in Midlife and Older, and we will use the acronym AIM to refer to the intervention. The AIM intervention has 2 components: an empowerment component and a social support component. These components are theoretically congruous with the framework we are using based on the empowerment model by Dutton [37] and the social support theory by Cohen [38]. We expect that the combination of the empowerment and social support components will be highly effective.

#### Components of AIM

The empowerment component is derived from the empowerment model by Dutton [37] to increase women’s safety. This component will involve the facilitator providing a 1-hour-long individual one-on-one information-sharing session with the participants that will be delivered virtually by telephone or video call. These sessions will include providing information on recognizing increased danger and how to use an individualized safety plan adapted for older women, providing information about cycles of violence, providing community and legal resources, and developing goals and strategies for the future. In addition to discussing this information, print materials will be provided electronically (via email) or in hard copy (via postal mail). Thus, this component clearly contributes to awareness raising and also empowers the women to develop changes in the future.

The social support component is based on the social support theory by Cohen [38] that involves tangible and perceived social support to contribute to health and well-being. This component will involve the facilitator scheduling weekly telephone or video calls over a course of 12 weeks at a time that is convenient to the participant. The duration of calls is variable and would average approximately 20 minutes per call. The calls are intended to provide encouragement and support and to answer questions the participant may have about using or applying the resources provided in the empowerment component. There are no specific or scheduled topics that are planned for each session. These are very individualized and based on the women’s needs and questions that each woman may wish to discuss. For example, facilitators will be prepared to help women with recognizing their past abuse and processing the abuse as well as providing support to women who are undergoing court proceedings and legal issues. The social support provided through the weekly contact is a key component of the intervention in addition to the topics discussed.

#### Comparison Group

The comparison group will receive a static, nontailored version of the AIM intervention materials after baseline data collection. They will be asked if they prefer to receive these materials via email or postal mail. The document will include a condensed version of the empowerment component and information about the dynamics of abusive relationship, warning signs of abuse for older adults, materials on safety planning and digital safety, local and national IPV resources, and guidance for setting goals for the future. The participants in the comparison group will not receive any further contact from the research team or facilitators to discuss these materials. Textbox 1 outlines the features (ie, information and support) that participants will receive depending on what group they are randomly assigned to.

**Features of the advocacy intervention for midlife and older women (AIM) intervention group and comparison group.**

**AIM intervention group**

- Receives the information package
- Participates in 1-hour-long information-sharing session with the program facilitator
- Participates in 12 weekly social support sessions with the program facilitator

**Comparison group**

- Receives the information package

### Sample Size

For the quantitative portion of the study (ie, the RCT), we determined an appropriate sample size that would be sufficient to contribute to the evidence base. We reviewed the sample sizes analyzed in the systematic review we conducted, the sample sizes of the studies focused on women experiencing IPV in midlife and older, and the sample size calculations in the study from which we have adapted the program [34]. We used our prior experience in recruiting midlife and older women as well as the resources available to conduct this research. We then concluded that recruiting 30 participants per arm is sufficient, that is, 30 women randomized into the intervention arm and 30 women randomized into the control arm. To account for a 15% attrition rate identified in a similar study [39], we will recruit 70 women, with 35 randomized to each group.

### Participant Recruitment

The facilitators were asked to recruit participants within their organization. Facilitators were encouraged to distribute the recruitment poster for the study to potentially eligible participants in the following formats: (1) through email, phone call, social media (eg, Facebook), or in-person visit or (2) through a poster placed in a central location (eg, a lobby of a building) or a handout given to potential participants. Potentially eligible women will be assured that choosing not to participate in the study will not negatively affect their access to other services. They will be asked whether they are comfortable with the facilitator providing the research team with their names and contact information, or alternatively, whether they would prefer to contact the research team on their own. Facilitators were asked to distribute the study’s recruitment poster throughout their region. This included sending email requests to organizations and distributing the poster to community bulletin boards.

Once a potential participant reaches out to the research team by phone or email or is referred by a facilitator, research staff (eg, research coordinator or research assistant) will respond and arrange a time to communicate with them by telephone. Research staff will provide an overview of the study details with each potential participant and answer any questions they have, ensure that they meet the study inclusion criteria, and review the informed consent forms. Once these first steps are complete, the research team will contact the participant to schedule a time to collect the baseline survey data.

We will include any woman who fulfills the eligibility criteria, and there are no quotas for women with specific characteristics, but we will try to include women from diverse backgrounds, including francophone women, new immigrants, women from visible and racialized minority groups, women living in rural places with limited access to services, and women in same-sex relationships. We will try to recruit women from diverse backgrounds by contacting organizations that offer services to women from marginalized or diverse backgrounds in the Maritime provinces and asking them to take part in a research partnership. Given that the professionals working in these organizations (eg, transition houses, outreach programs, and women’s centers) have the expertise in working with these populations, they will be instrumental in helping us to reach and recruit diverse women into the study. In this study, our definition of rural is informed by the rural and small-town definition, which includes towns with population <10,000 inhabitants and people who live outside the commuting zone of a large urban center (eg, >40 minutes) [40,41].

### Partnerships With Community-Based Organizations

#### Overview

The facilitators were recruited by the research coordinator with the support of the study coinvestigators and study partners. The study team developed a contact list of potential organizations throughout the Maritime provinces that deliver services for older adults experiencing abuse and those who work with women experiencing IPV. The organizations included transition houses, IPV outreach services, women’s shelters, women’s centers, and other service providers for women (eg, sexual health centers and addiction services). The research coordinator contacted the organizations via email to inquire about forming a partnership with the research team. The organizations were asked if they would be willing to collaborate with the study team by agreeing to 1 of the 2 research partnerships we offered. Organizations that expressed interest in partnering upon our initial email were requested to meet with the research team virtually to discuss the study and answer any questions.

#### Partnership 1

Organizations were asked if they would be interested in partnering with the study by appointing a staff member who could serve as a facilitator on the project. Organizations were notified that the facilitators would need to take part in a 2-hour training session on how to deliver the intervention and would be assigned the tasks of recruiting potential participants and delivering the intervention to any participants that they recruited within their organization. Organizations were informed that they would receive a stipend of CAD $2500 (CAD $1=US $0.74) for agreeing to partnership 1 and having a staff member take part in the training and recruiting and delivering the intervention to 1 to 5 participants. An additional CAD $2500 stipend will be offered to any organization that has a facilitator who recruits and delivers the intervention to ≥6 participants, given the increased time and resources this would take. A technology fee of CAD $1000 will be provided to each partnering organization for overhead costs and the use of their equipment (ie, laptops and phones) to deliver the intervention. Organizations were notified that staff members need to meet the following criteria to be eligible to become a facilitator: (1) at least 2 years of experience working in the IPV service delivery field and (2) completion of a postsecondary education program (eg, social work, health professions, human services, counseling, health promotion, or social sciences).

The research team also hired staff facilitators throughout the Maritime provinces to deliver the intervention to participants that were not recruited from within community-based organizations. The staff facilitators will be completing the same tasks but will be working independently (ie, not with an organization). All staff facilitators need to meet the minimum educational and experience requirements.

#### Partnership 2

We offered a CAD $500 honorarium to organizations that were willing to help us out with participant recruitment only. The research team asked partnering organizations if they could help with recruitment by distributing the recruitment poster to potential participants and completing 1 of the following tasks: (1) asking potential participants within their organizations for permission for a member of the research team to contact them and (2) share information about the study with potential participants who can contact the research team directly if they wish to do so.

### Training for Facilitators

All facilitators will take part in a virtual training session individually or in a group. The content of the AIM intervention was developed by LW, CM, and HH. A slideshow was developed by LW and provided to the rest of the study team for review. The slides were then translated to French to provide the training to francophone facilitators. Given that New Brunswick is a bilingual province, the research team wanted to ensure that we could recruit both anglophone and francophone participants in all regions in the province. The research coordinator scheduled the training sessions and provided a copy of the slides to the trainees before their scheduled training session. Textbox 2 provides an overview of the content that was included in the training slideshow.

**Facilitator training content.**

**Section 1: an overview of the advocacy intervention for midlife and older women (AIM) study**

- Study rationale
- Study goal and research questions
- Empowerment component
- Social support component
- Tasks to be completed
- Eligibility criteria for participants
- Study timeline
- Research team members

**Section 2: diverse midlife and older women and intimate partner violence**

- Applying an intersectional lens to violence against midlife and older women
- Intersectionality wheel
- Using a trauma-informed approach
- Wheels of power and control
- Warning signs that an older adult may be in an unhealthy relationship
- Working with rural women who are in midlife and older
- Working with Black women who are in midlife and older
- Working with midlife and older women who are immigrants
- Working with midlife and older women who are francophone

**Section 3: safety planning for older women**

- Safety planning toolkit
- Digital safety strategies: computer safety
- Digital safety strategies: cell phone safety

**Section 4: local community and legal resources**

- National resources
- New Brunswick resources
- Nova Scotia resources
- Prince Edward Island resources

**Section 5: supporting midlife and older women to develop goals and strategies for the future**

- Using specific, measurable, achievable, relevant, time-bound (SMART) goals

**Section 6: documenting the delivery of the AIM program**

- Plans for documenting sessions with participants on the study’s OneDrive

LW will be delivering the training session along with the research coordinator who will be present to answer any questions about the study procedures. The research coordinator will be responsible for communicating with the facilitators for the duration of the study. The training session was not designed to be a lecture but rather to share information and to have an open dialogue with the service providers who already have a great deal of experience providing services to clients. The training session was used to ensure that facilitators were informed on how to deliver the components of the AIM intervention in a highly individualized way. Facilitators were also encouraged to contribute to the list of resources provided if they had knowledge of other resources in their region that were not listed. Facilitators were informed that they could begin recruiting participants upon completion of the training session.

### Random Assignment of Interventions

REDCap (Research Electronic Data Capture; Vanderbilt University) software will be used to store and manage the data collected from participants. REDCap is a web-based software application that securely stores research data [42]. The random allocation sequence was generated using REDCap [42]. The research coordinator developed an allocation table that included each option for random assignment that was stratified by the province of residence. This involved creating an Excel (Microsoft Corp) table with each intervention option (ie, intervention and control) in one column and province of residence (ie, New Brunswick, Nova Scotia, and Prince Edward Island) in another column. We wanted to ensure that an equal number of participants were randomized to the intervention and control group within each province in a balanced manner. Therefore, there were 6 study options uploaded to the software. Once the allocation table was uploaded to REDCap, it was locked and could not be modified.

REDCap will be used to randomize the participants. After a participant has completed the baseline surveys, the researcher will press the “randomize” button to determine the participant’s group allocation. While assignment to the groups is random, once assigned, participants are not blinded to their group. The researcher will inform each participant of the group they are allocated to at the end of the baseline interview. The research staff (research coordinator and research assistant) will be responsible for screening, consenting, enrolling, and using Recap’s randomization module to allocate participants to their study group.

### Outcomes Measured

Research staff will collect quantitative effectiveness data from participants by telephone or video call at 3 time points: baseline, 3 months, and 9 months. Research staff will schedule a meeting by telephone or Zoom (Zoom Video Communications) to collect the survey responses from participants verbally. Table 1 details the survey information to be collected directly from participants.

**Table 1:** Quantitative measures.

Quantitative measure collected	Time point for data collection			Explanation
	Baseline	3 months	9 months	
Screening questions	✓			Age, province of residence, and description of the stage of their relationship with their abusive partner
Demographic information	✓			Education, marital status, and diversity characteristics (eg, immigrant status, francophone status, ethnic status, and rural status)
WEB^a^	✓	✓	✓	To measure women’s experiences of feeling fearful and controlled by their abusive partner [43]
DCS^b^	✓	✓	✓	To measure the conflict (ie, difficulty) that women endure in making decisions about leaving their abusive relationship [44]
IPV^c^ strategies index	✓	✓	✓	To measure the different types of IPV strategies that women are using to keep themselves safe in their abusive relationship [45]
SF-12v2^d^	✓	✓	✓	To measure quality of life [46]
CES-D^e^	✓	✓	✓	To measure depressive symptoms [47]
ISEL^f^	✓	✓	✓	To measure perceived social support [48]

^a^WEB: Women’s Experiences of Battery Scale.

^b^DCS: Decisional Conflict Scale.

^c^IPV: intimate partner violence.

^d^SF-12v2: Short Form Health Survey.

^e^CES-D: The Center for Epidemiologic Studies Depression Scale.

^f^ISEL: The Interpersonal Support Evaluation List.

Primary outcomes include (1) physical and mental health, (2) knowledge about IPV, and (3) safety strategies. Physical and mental health will be assessed using the following scales: Short Form Health Survey [46], Centre for Epidemiologic Studies Depression Scale [47], and Interpersonal Support Evaluation List [48]. Knowledge about IPV and safety strategies will be measured using the following scales: the Women’s Experiences of Battery Scale [43], Decisional Conflict Scale [44], and Intimate Partner Violence Strategies Index [45].

### Participant Involvement by Group

Participant recruitment began in October 2023 and is expected to continue until July 2024. Participants will complete the screening and informed consent process with research staff and will be randomized upon completion of their baseline surveys. Participants that have been randomized to the intervention group will be matched with a facilitator in their region. The facilitators will follow up after randomization and schedule the 1-hour-long information-sharing session (ie, empowerment component) and then continue to follow up with the weekly calls for 12 weeks (ie, social support component). Quantitative survey data will be collected from all the participants at 3- and 9-month follow-ups. Follow-ups with participants in the AIM intervention group will take place 3 and 9 months after their final social support session. For participants randomized to the comparison group, follow-ups will take place 3 and 9 months after they have completed the baseline surveys. We estimate the total time commitment for participants to be 11 hours for those randomized to the AIM intervention group and 4 hours for those in the comparison group. Table 2 breaks down the estimated time commitment for each study activity.

**Table 2:** Participant involvement by group.

Study activity	Time commitment for participants in the AIM^a^ intervention group (hours)	Time commitment for participants in the comparison group (hours)
Informed consent process	0.5	0.5
Baseline data collection	1.5	1.5
Empowerment component	1	—^b^
Social support component	6	—
3-month follow-up	1	1
9-month follow-up	1	1
Total time commitment	11	4

^a^AIM: advocacy intervention for midlife and older women.

^b^Did not participate.

### Data Management

REDCap software will be used to store and manage the study data [42]. The only individuals with access to the REDCap data will be the research coordinator and the research assistants. The research coordinator and the research assistants will enter the participants survey responses directly into the REDCap forms at baseline and 3- and 9-month follow-ups.

### Statistical Analysis

Quantitative data will be exported from REDCap as a spreadsheet and then uploaded into SPSS (IBM Corp) for quantitative analysis. Participants in the AIM intervention group need to complete the empowerment component and attend at least 10 out of 12 weekly calls of the social support component for their quantitative data to be included in the analysis.

Descriptive statistics will provide a profile of study participants, including demographics for the whole sample and for those in each of the 2 groups. The provisional effect of the intervention will be tested using a prospective chi-square test of independence between the 2 groups for categorical data (Fisher exact test) at 3 and 9 months. Repeated measures analysis of variance will measure mean differences between the groups on continuous variables at 3 and 9 months.

### Qualitative Component

We will conduct interviews with approximately 12 participants of the AIM intervention group. This sample size is sufficient when using a qualitative descriptive approach to generate rich, detailed descriptions and meaningful data [49,50]. In these interviews, we will use maximum variation sampling to include women who have specific characteristics (eg, francophone, new immigrant, and rural) and ask them about their perceptions of the program and their recommendations for further program development. We will also invite any intervention group participants who did not complete the AIM program to participate in an interview to learn about their experiences in the program and ask for any recommendations they may have to help improve the program. All facilitators who deliver the AIM intervention to trial participants will be invited by telephone or email by the research coordinator to complete a qualitative interview. While all will be invited, we hope that at least 4 to 5 will volunteer to participate in the interview.

At the 9-month follow-up data collection point, the research coordinator will invite women from the AIM intervention group to take part in a virtual interview. To be eligible to be invited for the interview, participants must have completed the empowerment component and at least 10 out of 12 social support sessions. In these interviews, the research coordinator will try to recruit a diverse sample of women who represent the characteristics of the participants and ask them about their perceptions of the program and their recommendations for further refinement of the program to meet the needs of diverse midlife and older women. The informed consent form will be shared by email or by postal mail.

The research team will also conduct an exploratory qualitative analysis on the data recorded by the program facilitators. Facilitators will be instructed to record the date and length of each session and take notes. The notes will detail the topics discussed with participants, the support and resources offered to participants, and any questions or concerns raised by the participants. The analysis of this exploratory data will summarize what topics are discussed and what supports are provided to participants.

All qualitative data will be analyzed using thematic analysis [51]. This is a form of pattern recognition allowed for inductive coding [52] and is particularly useful in understanding the influences and motivations related to how people respond to events [53]. The data coding process will involve generating initial codes, searching for themes, reviewing themes, and defining and naming themes that result in thematic codes representing patterned responses within the dataset [51]. Qualitative interview data from the participants of the intervention group will provide important insights about further refinement of the program to meet the needs of diverse women. Qualitative interview data from facilitators will provide important insights about the training program they participated in, delivery methods and content of the program to consider in further development, and scale-up and spread of the program.

### Ethical Considerations

#### Overview

This trial required research ethics approval from the institutions where the investigators are located, which included Dalhousie University, the University of New Brunswick, St. Francis Xavier University, the University of Prince Edward Island, and the Université de Moncton (Table 3). To facilitate this process, the application was first submitted and approved by Dalhousie University’s Social Sciences and Humanities Research Ethics Board and then subsequently submitted to the other research ethics boards. The trial was deemed more than minimal risk and, therefore, underwent a full, rigorous review at 4 of the 5 institutions. St. Francis Xavier University conducted an expedited review because the trial was previously approved by Dalhousie University.

**Table 3:** Institutional review board approvals.

Institutional ethics review board	Date of approval	REB^a^ file number
Dalhousie University Health Sciences Research Ethics Board	July 11, 2023	2023-6548
St. Francis Xavier University Research Ethics Board	August 23, 2023	26598
Université de Moncton Ethics Committee for Research Involving Human Beings	September 6, 2023	2324-005
University of New Brunswick Research Ethics Board	September 23, 2023	2023-110
University of Prince Edward Island Research Ethics Board	October 6, 2023	6012156

^a^REB: research ethics board.

#### Informed Consent Process

All potential participants will be provided with information about the study and will receive either an electronic or a mailed copy of the informed consent form and will be given an opportunity to review it and ask questions before deciding whether they wish to volunteer to participate. The consent form will be reviewed verbally with the potential participant, and verbal consent will be obtained before beginning data collection. The person collecting the data will electronically record that consent was obtained before the first data collection point and before the 2 follow-up data collection points. After consenting to take part in the study, the participants and research staff will agree on a time to conduct the baseline interview by telephone or video call. At the beginning of each follow-up interview, key components of the consent form will be reviewed, and any questions raised will be responded to before the interview proceeding.

Participants can choose to withdraw by canceling the scheduled data collection session, withdrawing at the beginning of the session, or withdrawing after the data collection session begins. Participants can withdraw their data up to 7 days after a data collection session. This information is included in the consent form, and this will be emphasized during the review of the consent form before data collection begins.

#### Confidentiality and Anonymity

All study data will be kept confidential, and the participants’ identity (eg, names and identifying information) will not be made known in any reports of the results. A participant ID number will be used for any quotes in reporting qualitative results. Qualitative interviews will be recorded on the research coordinator’s secure laptop. The transcription function in Microsoft Word will be used to create a draft transcript that will then be edited by the research team members. Once a transcript is checked and edited by a research team member, the interview transcripts and the quantitative data will be shared among the team members using OneDrive, a secure, password-protected file-sharing system hosted by Dalhousie University. No hard copies of any study documents that could identify a participant will be retained.

The digital recordings will be kept only until the transcript is prepared, and then, the recording will be destroyed. Qualitative data, numerical key codes, and contact information of the participants will be retained by the co–principal investigators on a password-protected computer and on the OneDrive systems at Dalhousie University and the University of New Brunswick. These data will be destroyed 5 years after the publication of study results. Each participant will be given the option of allowing the co-principal investigators to retain the deidentified quantitative data indefinitely to be used in potential future research studies or for teaching purposes (eg, in a course with content on family violence). Providing this consent is not required for inclusion in the study.

This study focuses on midlife and older women who are currently experiencing IPV and are either living with an abusive partner or are in the process of leaving an abusive partner. There are different laws regarding the duty to report elder abuse in different provinces where we will be collecting data, and we will follow the procedure in each province. However, the issues about when to report typically are focused on the situations in which people do not have the physical or mental capacity to protect themselves or make their own decisions. If in the process of conducting data collection, the abuse of a child or an adult in need of protection is disclosed, the facilitator will contact the appropriate child protection or adult protection services in the location where the data are being collected.

The participants could be clients of an agency the facilitators work for. The participants will be assured that whether they choose to participate in the AIM study or not, there will be no negative repercussions on other services they access.

#### Compensation

All participants in the quantitative study will be provided with an honorarium of CAD $125 after completion of their baseline surveys. The honorarium is provided for agreeing to take part in the study and is not provided as an incentive. An additional CAD $20 honorarium will be provided to those who participate in a qualitative interview. All facilitators will receive an honorarium of CAD $45 if they participate in a qualitative interview. All participants will be given the option of receiving a physical gift card sent in the mail or a digital gift card sent via email.

#### Potential Harms to Participants

For participants living at home with the perpetrator, the participant may be at risk if the perpetrator finds out they are participating in the study. We will ensure personal safety of the participants in various ways at each stage of the research, including participant recruitment. The participants are welcome to participate virtually at a time and location that is chosen by them. If they need to end participation at any time, they are welcome to do so, and the facilitator will follow up at a time that is suitable and safe for them. As both the empowerment and social support components are delivered individually to participants, we can be very flexible about when the intervention is delivered.

The facilitators will teach the participants about ensuring digital security (eg, clearing browser history) during the empowerment component of the intervention. The participants will also be provided with information about local resources and services that are available. If the participant is in immediate danger during the time the facilitator is talking with them, they will instruct the participant to call 911, and if that is not possible, they will call 911 on the participant’s behalf.

#### Data Monitoring

This pragmatic RCT does not have a data monitoring committee. Participant safety will be monitored through the research coordinator’s regular communications (eg, biweekly communication when the facilitators are delivering the intervention) about any potential harm to the participants. However, we do not expect that the intervention will cause harm to the participants. The main potential risk that we identified is for the participants currently experiencing abuse and potentially living at home with the perpetrator. The perpetrator may find out about the study and may be against participation. The participants can leave the study at any time, and they have been provided with information about local resources and services that are available.

### Access to Data

The data from this trial will not be publicly available due to the risk of compromising the privacy of the research participants.

### Dissemination of Results

The findings of this study will be disseminated in several ways. We will submit a journal article to a peer-reviewed journal (eg, *Journal of Women and Aging* and *Violence Against Women*). We will ensure that the article is open access to ensure accessibility. We will deliver conference presentations at academic and professional conferences (eg, Canadian Association on Gerontology Annual Conference and Canadian Domestic Violence Conference). The intended audience will be researchers, practitioners, students, trainees, and policy makers. We will also develop and disseminate other research outputs that are required by the funders (eg, plain language summary, brief video, and infographic) in English and French. These accessible documents will be important for all our team members to share with their networks.

If the AIM intervention is determined to be effective, we will use the feedback we receive from the qualitative interviews with staff and participants to refine the staff training program and make it publicly available in both French and English. The final program materials will be distributed to service providers who work with victims of IPV and elder abuse in the Maritime provinces. In addition, the final program will be made freely available to anyone interested in supporting midlife and older women experiencing IPV and will be posted and publicized in the resources section of the Muriel McQueen Fergusson Centre for Family Violence Research website. The program will also be posted and distributed through the resource hub of the Canadian Network for the Prevention of Elder Abuse.

## Results

The funding from the Public Health Agency of Canada for this study began in January 2023. From January to June 2023, the initial ethics proposal was drafted and research staff were hired. Research ethics applications were submitted to each university from July to October 2023. Table 2 provides the research ethics board approval numbers for each institution. Recruitment of facilitators began in July 2023 and continued till May 2024. The principal researcher conducted 5 AIM training sessions with 12 facilitators from the Maritime provinces. Four of the training sessions were conducted in English only, and 1 training session was provided to a francophone facilitator via Zoom using simultaneous translators. Participant recruitment and data collection will be carried out from November 2023 to January 2025. Data analysis will continue throughout the data collection period, and results will be disseminated by December 2025.

## Discussion

### Anticipated Principal Findings

We anticipate that the quantitative data will reveal positive outcomes for participants in the intervention arm on our primary outcome measures of (1) physical and mental health, (2) knowledge about IPV, and (3) safety strategies. Given that the participants in the control arm will receive a static, nontailored version of the AIM intervention materials after baseline data collection, we expect that there could be some positive outcomes on items such as knowledge about IPV and safety strategies but to a lesser extent than for the participants in the intervention arm.

We anticipate that the qualitative data will reveal the value of offering the AIM intervention to midlife and older women who have experienced IPV. We expect that the qualitative findings will reveal how the content and delivery of the program can be improved for both the participants and the facilitators.

### Limitations

The results of this trial may not be generalizable to the larger Canadian context given the smaller, predominantly rural, population in the Maritime provinces. Another limitation to this study is that it relies on self-reported data from the participants.

### Comparison With Prior Work

This study represents a novel intervention that is urgently needed to support midlife and older women who experience IPV in the Maritime provinces of Canada. Our previous research indicated that initiatives focused specifically on supporting older women who experience IPV are lacking [14], and diverse older women who experience IPV need additional supports [15]. The AIM study is novel in the following ways: (1) the AIM intervention was adapted to focus on the specific needs of diverse midlife and older women that experience IPV whose needs are often ignored by service providers and (2) the intervention is entirely virtual, which will reduce barriers to participation.

### Directions for Future Research

Future research is needed to examine the implementation of the AIM intervention after adaptations are made, based on the findings of this RCT, preferably in a pan-Canadian study.

### Conclusions

This research will result in the adaptation and testing of a program to support and empower diverse midlife and older women who experience IPV. This research will significantly enhance our understanding of a population that has been historically understudied and underserved. The quantitative findings from this RCT will provide preliminary evidence about the effectiveness of the intervention. Interview data from the participants in the intervention group will provide important insights about further refinement of the program to meet the needs of diverse midlife and older women. Interview data from facilitators will provide important insights about the training program they participated in, delivery methods and content of the program to consider in further development, and scale-up and spread of the program.

## Abbreviations

AIM: advocacy intervention for midlife and older women
CONSORT: Consolidated Standards of Reporting Trials
IPV: intimate partner violence
RCT: randomized controlled trial
REDCap: Research Electronic Data Capture
SPIRIT: Standard Protocol Items: Recommendations for Interventional Trials

## Data Availability

The datasets generated and analyzed during this study are not publicly available due to the sensitive nature of this research and the participants not providing consent for their data to be shared publicly.
